# Examining the effects of a modified SART when measuring mind‐wandering

**DOI:** 10.1002/brb3.3175

**Published:** 2023-07-20

**Authors:** Yuqing liu, Qin Dong, ChengHao Yu, YuTong Jin, ChenYuan Fang, Yu Hu, Qiang Zhou

**Affiliations:** ^1^ Department of psychology Wenzhou Medical University Wenzhou Zhejiang China; ^2^ Key Laboratory of Alzheimer's Disease of Zhejiang Province, Institute of Aging Wenzhou Zhejiang China; ^3^ Psychology and Behavior Research Center, Wenzhou University Wenzhou Zhejiang China

**Keywords:** cognition, mind‐wandering (MW), SART performance, thought probe

## Abstract

**Objective:**

Mind‐wandering (MW) is defined as a shift of attention from external tasks toward internal thoughts and is popularly measured by the sustained attention to response task (SART). SART is able to capture MW, but cannot track the dynamics of mind‐wandering over time well. We thus attempted to modify the sustained attention to response task paradigm (mSART) to capture the participant's mind‐wandering state over time and quantify the degree of mind‐wandering using the current behavioral data.

**Methods:**

179 participants from Wenzhou Medical University were recruited to participate in this experiment. The main changes to the experiment included (1) manipulating different no‐go stimuli frequencies to control the difficulty of the task and setting 9 modes; (2) extending the experiment time to 30 min; (3) allowing participants to correct errors by pressing the b key. Error rate, Mean RTs, RT *CV*, and d' were used to reflect MW. Analysis of covariance (ANCOVA) was performed.

**Results:**

ANOVA was used to explore Mean RTs, RT *CV* and d’ for participants with different levels of mind‐wandering and significant differences were found (Mean RTs:Welch's *F* (2, 8606.04) = 579.00, *p* < .001, *η_p_^2^* = 0.03; RT *CV*:Welch's *F* (2, 198.11) = 69.93, *p* < .001, *η_p_^2^* = 0.18; d':*F* (2, 176) = 19.88, *p* < .001, *η_p_^2^* = 0.18). The 30‐min experiment was divided into six time windows, and mind‐wandering deepens over time.

**Conclusions:**

The mSART paradigm could quantify the extent of MW based on changes in the frequency at which the no‐go stimuli were presented and also revealed that the recommended length of the experiment was about 20 min.

## Graphical abstract

We modified the original SART paradigm from a single task with fixed stimuli frequency to one in which the difficulty of the task was regulated by adjusting the frequency of no‐go stimuli and linking participants' error rate and reaction time to the degree of mind‐wandering, thus achieving clarity of mind‐wandering through current behavioral data alone. The result showed that mSART not only did not change the simple structure of the original paradigm, applicable to participants with lower cognitive levels, but also effectively captured mind‐wandering over time.

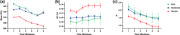

## 1 INTRODUCTION

The sustained attention to response task (SART) was first proposed by Robertson et al. (1997) to assess sustained attention over a short period. It is a simple go/no‐go task, where digits ranging from 1 to 9 are usually selected for the stimuli condition (e.g., a go stimulus represented by digits 1 to 9, excepting 3; a no‐go stimulus represented by 3) (Baldwin et al., 2017; Durantin et al., 2015; Jonkman et al., 2017; Martínez‐Pérez et al., 2021). Early paradigms for measuring sustained attention were based on resource control theory and typically required participants to monitor long stimuli sequences and respond with key press when low‐frequency go stimuli were present. Robertson et al. (1997) argued that this was very susceptible to rapid automation and proposed adjusting the frequency of go stimuli and no‐go stimuli so that participants would need to consistently respond to go and not respond when low‐frequency no‐go stimuli were presented, which was the primitive SART paradigm. In this task, participants were asked to maintain a balance between response speed and accuracy; they must press the “j” key to response to go stimuli, but do not to no‐go stimuli (i.e., digit 3).

SART was initially used to measure the sustained attention abilities in brain‐injured patients and has since been widely used in psychiatric disorders such as schizophrenia, depression, attention deficit, and hyperactivity disorders (Bellgrove et al., 2005; Chan et al., 2004; Farrin et al., 2003). In addition, it has also been applied in mind‐wandering (MW) studies due to its simple and nonarousing structure, which allows participants to engage in various cognitive activities during the task, including nontask related thoughts (Hawkins et al., 2019). Mind‐wandering (MW) is defined as a shift of attention from external tasks toward internal thoughts, resulting in competition for the limited cognitive resources available for primary tasks (Arabaci & Parris, 2018; Smallwood & Schooler, 2006; Smallwood et al., 2007). MW is a pervasive and ubiquitous mental phenomenon, and individuals may spend up to about 50% of their waking time in this state (Chaieb et al., 2019; Kane et al., 2007; Killingsworth & Gilbert, 2010). While some previous studies have focused on the negative effects of mind‐wandering (Mooneyham & Schooler, 2013; Smallwood & Schooler, 2015; Yamaoka & Yukawa, 2020), such as distracting the participant (Feng et al., 2013) and its correlation to negative emotions and poor mental health (Bozhilova et al., 2018; Chaieb et al., 2019; Guesdon et al., 2020; Jonkman et al., 2017; Lanier et al., 2021; Mowlem et al., 2019; Poh et al., 2016), numerous studies have also demonstrated that mind‐wandering has positive effects, such as improving creativity and problem‐solving (Agnoli et al., 2018; Gable et al., 2019; Leszczynski et al., 2017; Tan et al., 2015; Yamaoka & Yukawa, 2016, 2017), and future planning (Baird et al., 2011; Smallwood et al., 2009).

It is worth mentioning that a series of measures have been developed to investigate mind‐wandering (Robison et al., 2019), including subjective/direct and objective/indirect methods, such as questionnaires (e.g., Bozhilova et al., 2018; Jin et al., 2019; Frewen et et al., al.,^2020^; Stawarczyk et al., 2020), retrospective reports following a task (e.g., Antrobus et al., 1966), and cognitive activity performance (e.g., Zheng et al., 2019; Voss et al., 2018; Hutchison et et al., al.,^2020^). However, self‐report method, including self‐capture and probe capture, has been the most commonly used method in recent years (Weinstein et al., 2018a). The self‐capture type requires participants to remain aware of their internal states throughout the task without any reminders to do so; under this condition, they will immediately report any experiences of MW. The probe capture type involves stopping participants during a task, presenting them with a thought probe, and asking them to describe their thought‐state (Weinstein, 2018b).

The initial SART paradigm structure itself could quantify the overall performance of participants in the task but might not profile the dynamics of MW over time. Specifically, we found that there was a gradual occurrence of response error to no‐go stimuli during the task and that participants sometimes became aware of their response errors and chose to adjust their manual responses (Cheyne et al., 2009). With present behavior data alone, it is difficult to know the extent of their MW state at a specific moment, and when this state starts, ends, and how it changes over time. Surely, inserting probes into the task can help capture the participants' mind‐wandering state at specific time windows. However, it has been increasingly proposed that probe‐caught method limited the instances and the locations where mind‐wandering could be detected, for example, by interrupting their present state of awareness (Kopp et al., 2015). Seli et al. (2013) noted that the frequency of probe presence can significantly affect the reported mind‐wandering, with the longer the interval between probes, the higher the reported mind‐wandering frequency. Weinstein et al. (2018a) found that even when tasks are similar, subtle differences in the wording of probes and response options may affect self‐reported mind‐wandering rates. Furthermore, the differential impact of probes on individuals is also an issue (Wiemers & Redick, 2019). In general, the original SART paradigm is unable to track participants’ MW changes over time, but the influence of probes can no longer be ignored. Thus, we wondered if this could be achieved by changing the structure of the SART itself.

The simple and rote nature of the SART makes MW episodes during performance are frequent (Smallwood et al., 2004). Given this, mind‐wandering was operationally defined: the inability to withhold responses to no‐go stimuli can be seen as evidence of failing to adequately attend to the task. Specifically, a manual response to an uncommon no‐go (an error) stimulus was a behavioral marker of mind‐wandering, whereas correctly withheld responses were considered “on‐task” (Cheyne et al., 2009; Smallwood et al., 2004). Reaction times (RTs) before the no‐go stimuli response errors were also related to task performance, with faster SART RTs directly reflecting the attentional lapses that led to SART errors (Manly et al., 1999; Robertson et al., 1997). Hence, SART RTs and SART errors were used to reflect the performance of the task and MW.

Based on this, we attempted to modify the SART paradigm (mSART) to dynamically capture the participant's mind‐wandering state over time and quantify the degree of mind‐wandering. Specifically, we changed the original SART paradigm from a single task with fixed stimuli frequency to one in which the difficulty of the task was regulated by adjusting the frequency of no‐go stimuli and linking participants' error rate and reaction time to the degree of mind‐wandering, thus achieving clarity of mind‐wandering through current behavioral data alone.

## 2 METHODS

### 2.1 Participants and design

Using G*Power 3.1.9.7, a minimum of 159 participants was identified as being required to conduct the study, with a maximum effect size of 0.25, an alpha level of 0.05, and power of 0.80. Sample size based *F*‐test for one‐way ANOVA with within‐subject design, effect size was choose from the study of Martínez‐Pérez et al. (2021) and Polychroni et al. (2020). Students from Wenzhou Medical University were recruited to participate in this study. A total of 185 participants (*M* = 20.281 years, *SD* = 0.547) with normal vision or corrected vision and no mental illness were recruited, consisting of 123 females (*M* = 20.171, *SD* = 0.455) and 62 males (*M* = 20.500, *SD* = 0.641). Following manipulation checks, we considered the following data as invalid: (a) the accuracy of both the no‐go and go stimuli was less than 5% (*n* = 2) and (b) the data are incomplete (*n* = 4). Thus, the sample in the final analysis included 179 participants, 6 of whom were excluded.

The within‐subject design was used in our experiment. All participants received the same mSART task programmed by E‐prime 2.0 software.

### 2.2 Design

The main settings and changes are as follows:

1. The fixed length of time for the SART paradigm: the length of the experiment has not been clearly defined and varies considerably in previous SART studies. Some researchers have used only 225 stimuli for approximately 5 min in their experiments (Bedi et al., 2023; Durantin et al., 2015), while others have used SART experiments with as many as approximately 500–900 stimuli for a total duration of approximately 16 min or longer (Baldwin et al., 2017; Jonkman et al., 2017; Seli et al., 2016a). We also noticed the fact that participants’ mind‐wandering did not always get deeper with increasing time, but generally reported they were MW‐ not MW‐MW, repeatedly (Cheyne et al., 2009), throughout the task, so we hypothesized that if the SART paradigm were used to induce mind‐wandering, then experimental time would be an important factor. Considering that past studies were within 20 min, we extended the time span to 30 min appropriately to compare the performance of the SART task across time intervals, so as to track the variation of mind‐wandering over time and explore the optimal length of time to induce MW.
2. The difficulty of the task was controlled by setting different no‐go stimuli frequencies, and the participants' mind‐wandering was measured by consecutive errors and reaction time. Mind‐wandering is considered present when participants consecutively respond to no‐go stimuli. Here, incorrect button responses indicate control failure for cognitive processing, meaning that attention is not focused on the task. In contrast, successful responses indicate that participants are focused on the task, and are thus without mind‐wandering. Specifically, we set up nine different modes (The probability of no‐go stimuli is 1/9, 1/9, 2/9, 3/9, 4/9, 5/9, 6/9, 7/9, 8/9), every time a mistake was made, it would enter the next mode. After successive errors to no‐go stimuli, the program continued to the next stage until the participant was removed from the mind‐wandering, and the program automatically returned to mode 1 after the correct no‐go response has occurred, and so on until the end of 30 min. It is worth mentioning that the go stimuli are highly predictable, which allows the participants' responses to go stimuli were highly automatic (Robertson et al., 1997), so our continuous error refers to the response error to no‐go stimuli.

The reason for setting 9 modes is that, in the SART paradigm originally proposed by Robertson et al. no‐go stimuli account for 1/9 of the total number of stimuli, and we planned to increase the probability of no‐go stimuli sequentially starting from 1/9. Mode 1 had the same no‐go stimuli probability as mode 2, due to the fact that a single response error to no‐go stimuli may be a random error that cannot be considered to be caused by mind‐wandering. It is evident that the SART paradigm can adjust task difficulty by manipulating the rate of no‐go stimuli; the higher the probability of no‐go stimuli, the less difficult the task, whereas if participants still make errors when the probability of no‐go stimuli was high, it was presumed that participants were in a deeper mind‐wandering degree. In other words, no‐go stimuli with higher frequencies were used to test whether and how deeply participants were mind‐wandering. In this context, they entered modes (1–9) with higher no‐go frequencies as the degree of mind‐wandering increased. We, therefore, calculated the deepest mode reached during the experiment and linked it to the different levels of mind‐wandering. Specifically, they were classified into three mind‐wandering categories, including mild (1–3), moderate (4–6), and severe (7–9).

3. Adding the “b” key: participants were told before the experiment that they needed to press the “b” key when they were not mind‐wandering but made a wrong keystroke, and then their program could return to the initial mode 1. This prevented some participants from making successive errors into a mode with higher no‐go stimuli rate because they could not inhibit their automatic responses, rather than mind‐wandering.

### 2.3 Procedure

**FIGURE 1:**
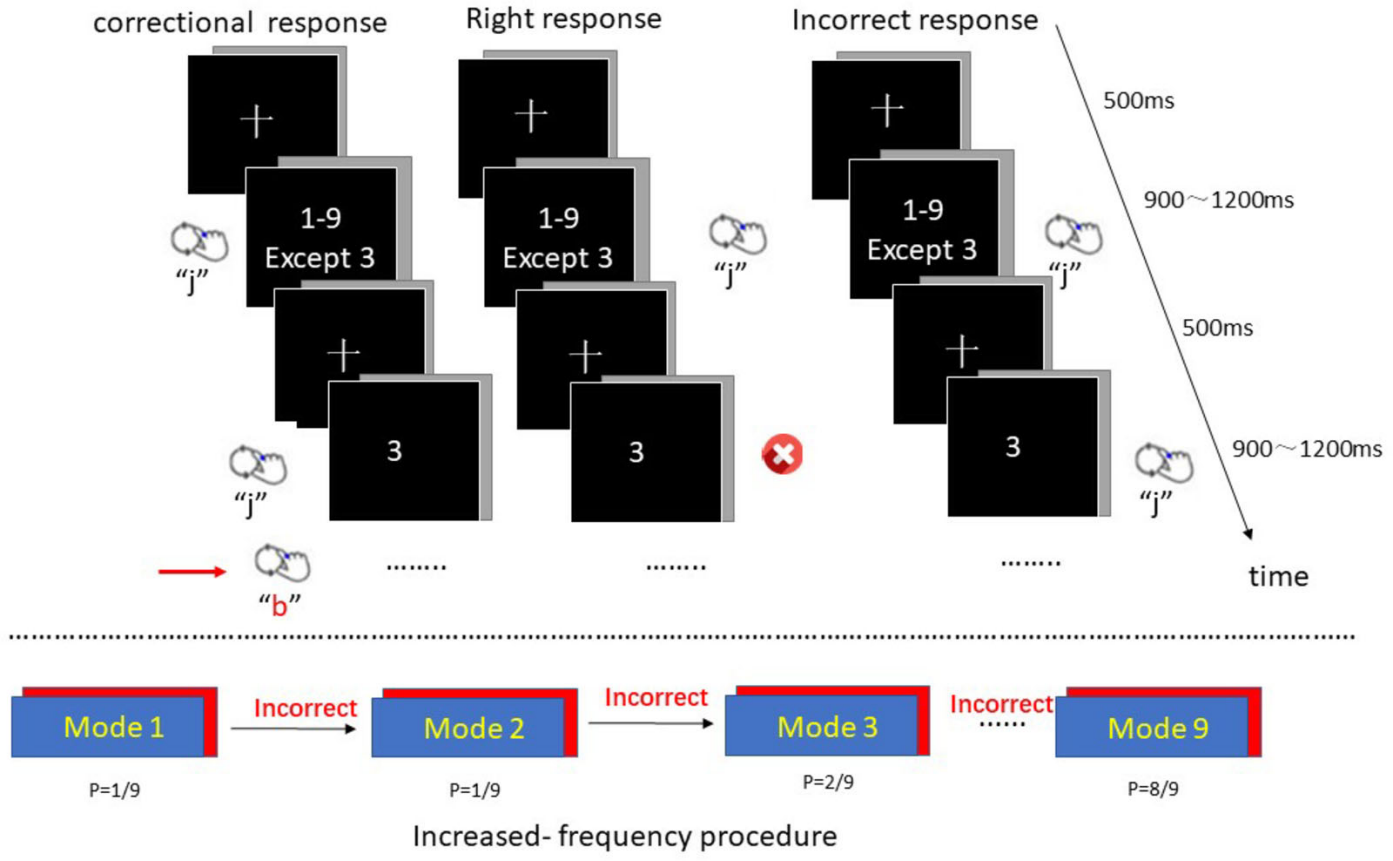
Experimental paradigm.

The experiment was conducted on a computer with a resolution of 1024×768 and a refresh rate of 60 Hz. Each participant was seated directly in front of the computer screen and completed task through E‐prime 2.0 software. All participants signed an informed consent form before the formal experiment. In the mSART task, participants were firstly presented a white cross in the center of the black screen (with a duration of 900–1200 ms), and then they were presented a single white digit on the black background (with a duration of 500 ms). The digit is selected from Arabic numerals 1–9, with a size of 96 pt, and the font is Calibri, which is also displayed in the center of the screen. The cross size and number font/size were unchanged. The experimental process is detailed in Figure 1.

Participants are required to press the “j” key to respond to all single digits except digit “3,” and withhold respond when digit “3” appears. The stimuli were randomly presented in a certain proportion to ensure that the participants could not predict their sequence. When they perceive their previous no‐go error, they can press the “b” key to correct or retract. To help participants understand and familiarize themselves with the experimental operation, we implemented 18 practice trials (including 2 no‐go stimuli and 16 go stimuli) before the formal experiment. In the formal experiment, we asked participants to respond as quickly and accurately as possible. The experiment was timed to automatically end after 30 min.

Experimental paradigm: there were three types of responses, including right, incorrect, and correctional responses. The experiment returned to mode 1 under a correctional response but continued to the next mode under an incorrect response. Specifically, participants entered mode 1 at the beginning and then (1) continued in mode 1 until the end of the 30‐min experiment if their no‐go response was correct. It is worth mentioning that during the experiment, if the no‐go response was correct after entering a certain mode, the participants would back to mode 1. (2) If participants responded to no‐go stimuli incorrectly, they would enter the higher mode, that is, a higher rate of no‐go stimuli. (3) If the participant pressed the wrong button by accident, they can correct the error by pressing the “b” key and then return to mode 1.

### 2.4 Definition and data analysis

#### 2.4.1 Definition

*Error rate*: key presses to no‐go stimuli were considered as no‐go stimulus errors, and their ratio out of all no‐go stimuli was the error rate of no‐go stimuli. Similarly, the no‐key press to a go stimulus was considered as a go response error, and its ratio to all presented go stimuli was the error rate of the go stimuli.

*Mean RTs and RT variability*: we calculated Mean RTs for the 4 go stimuli before the no‐go stimulus (Robertson et al., 1997). Coefficients of variability of reaction times (*RT Variability*; RT *CVs*) in the SART task were defined as the standard deviation of reaction times for the corresponding go stimulus divided by the mean (RT *CV* = *SD*/mean) (Cheyne et al., 2009). Referring to Cheyne et al. (2006), we excluded the 2 sets of RTs before the first 2 no‐go stimuli and RTs with fewer than 4 go stimuli between no‐go stimuli. Reaction time variation is an important indicator of inhibition function in brain's executive control system; higher RT *CVs* represent deeper degree of mind‐wandering.

*d‐prime (d')*: In signal detection theory the sensitivity index d' was calculated as the difference between the Z‐values of hit rate and false alarm rate. In this study, the proportion of correct no‐go trials was taken as hit rate, and incorrect go trials were taken as false alarms, and calculated the difference between the Z‐values of the two as the sensitivity index d' (Vermeiren & Cleeremans, 2012). A smaller d' indicates that it is less able to recognize the no‐go stimuli from the go stimuli, implying a deeper degree of mind‐wandering.

*Consecutive errors and deepest mode reached*: We defined it as starting to mind‐wandering when the participant responded consecutive errors to no‐go stimuli. If a participant entered the experiment and started to make mistakes, and after 5 consecutive error response to no‐go stimuli, he/she would enter mode 6. If he/she then started to respond correctly in the 6th time, task then returns to mode 1 and he/she was considered to be back in the task. The number of consecutive errors was 5, and deepest mode reached was 6. The higher the number of consecutive errors, the deeper mode is entered, defined as the more severe the mind‐wandering.

*Mind‐wandering classification*: A total of nine modes were designed for the experiment. We classified participants according to the statistics showing the highest modes entered during the experiment. As mentioned earlier, participants made more consecutive errors when they entered a deeper mode in a single round means that they made more errors in a row, indicating that they were more severely mind‐wandering. They were thus divided into three groups based on their respective levels of mind‐wandering, including mild, moderate, and severe. More specifically, participants who entered modes 1–3, 4–6, and 7–9 were considered to be mild, moderate, and severe mind‐wandering respectively.

*Time windows*: To explore and better quantify changes in the situation within 30 min and facilitate statistics, the time period was divided into six equal sections, each of 5 min, that is, 1–6 represented the time interval of the experiment process. For example, “1” represented 0–5 min, “2” represented 6–10 min, and so on; finally, “6” represented 26–30 min of SART task. The rate of stimuli errors during each of these time periods was calculated to explore the participants’ performance during the experiment. The error rate of go and no‐go stimuli in the six time intervals were analyzed and compared to explore the optimal time to induce mind‐wandering.

#### 2.4.2 Data analysis

All analyzes were conducted using IBM SPSS version 21.0. The following variables reflected participants' mind‐wandering: error rate, deepest mode reached, Mean RTs, RT *CV*, and d'.

**FIGURE 2:**
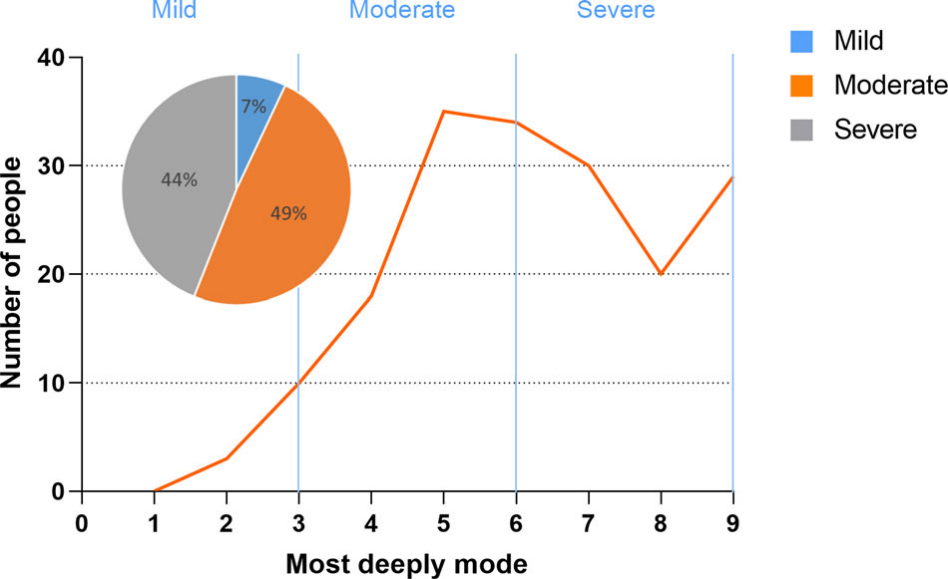
Participants’ mind‐wandering distributions.

First, descriptive data such as error rate, deepest mode reached, d', Mean RTs, RT variability for all participants were presented in Table 1. In addition, we categorized participants into three levels: mild, moderate, and severe, based on the deepest mode they reached in the experiment. Pie charts were used to depict the specific distribution of participants at each level (see Figure 2).

**TABLE 1:** The results of descriptive statistical.

	*M*	*SE*
Error rate		
No‐go stimuli	0.502	0.010
Go stimuli	0.083	0.005
Mean RTs (ms)	320.557	0.352
RT *CV*	16.955%	0.001
d’	1.506	0.023

**FIGURE 3:**
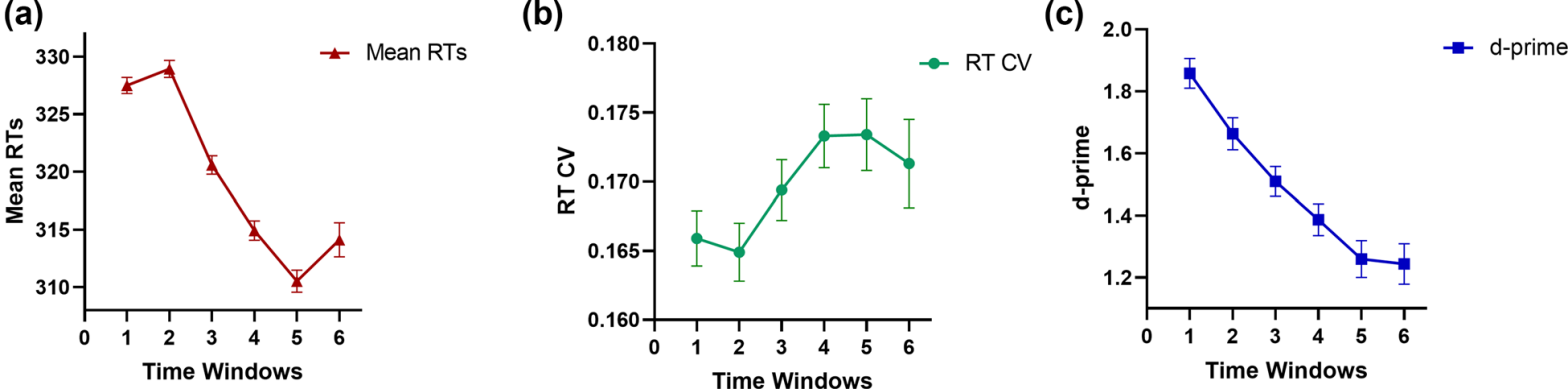
Participants' mind‐wandering during different time windows. Participants' mind‐wandering during different time windows. (a)–(c) represent how the participants' Mean RTs, RT CV, d’ changed over time.

**FIGURE 4:**
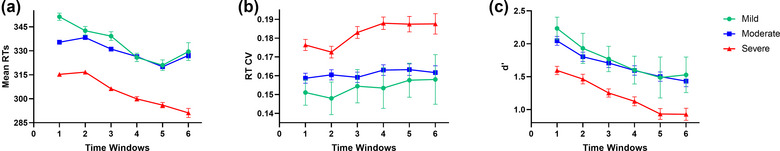
Three groups’ MW in different time windows. Three groups’ MW in different time windows. (a)–(c) represent how the participants' Mean RTs, RT CV, d’ changed over time.

Further, an ANOVA was used to compare the mind‐wandering in each time window, and Figure 3 presented the Mean RTs, RT *CV*, and d' for all participants in six different time windows. Several variables did not qualify for variance homogeneity in ANOVA, so Welch‐ANOVA analysis was used. In addition, we also analyzed the mind‐wandering of the three groups in the six time windows. Figure 4 presented the changes of Mean RTs, RT *CV*, and d' in the three groups in the different time windows. More detailed data were provided in Supplementary Materials 1 and 2.

## 3 RESULTS

To evaluate the effects of the mSART on mind‐wandering, the results were divided into three main sections: (a) the results of the mind‐wandering analysis; (b) time window analysis; and (c) “b” key. The descriptive statistical results of various dependent variable indicators were in Table 1.

### 3.1 Mind‐wandering analysis

We divided the MW degree into three groups using the deepest mode reached (Mild: deepest reached modes 1–3; Moderate: deepest reached modes 4–6; Severe: deepest reached modes 7–9). There were 13 mild (approximately 7%), 87 moderate (49%), and 79 severe cases (44%) (Figure 2). We explored Mean RTs, RT *CV*, and d’ for participants with different levels of mind‐wandering using ANOVA and found significant differences (Mean RTs: Welch's *F* (2, 8606.04) = 579.00, *p* < .001, *η_p_^2^* = 0.03; RT *CV*: Welch's *F* (2, 198.11) = 69.93, *p* < .001, *η_p_^2^* = 0.18; d': *F* (2, 176) = 19.88, *p* < .001, *η_p_^2^* = 0.18) (see Table 2). In terms of Mean RTs, post hoc comparisons revealed that Mean RTs in the mild group were greater than Mean RTs in the moderate group (*p* < .001), greater than severe group (*p* < .001), and Mean RTs in the moderate group were greater than in the severe group (*p* < .001) (i.e., Mild group > Moderate group > Severe group). For RT *CV*, post hoc comparisons showed significant differences between mild and severe (*p* < .001), moderate and severe (*p* < .001), except for the mild and moderate groups (*p* = .144) (i.e., Mild group = Moderate group < Severe group). For the d‐prime, post hoc comparisons showed that participants in the mild group were not significantly different from those in the moderate group (*p* = .643), but the mild group was larger than the severe group (*p* < .001) and the moderate group was larger than the severe group (*p* < .001) (i.e., Mild group = Moderate group > Severe group).

**TABLE 2:** The results of descriptive statistical under different MW classification.

	Mild		Moderate		Severe	
	*M*	*SE*	*M*	*SE*	*M*	*SE*
Mean RTs	335.750	1.207	330.320	0.450	306.730	0.582
RT *CV*	0.150	0.004	0.160	0.001	0.180	0.001
d’	1.770	0.195	1.690	0.058	1.210	0.051

Participant mind‐wandering distributions: the pie chart shows the proportions of participants in each group. The line chart reflects the specific distribution of the number of people's deepest mode reached.

### 3.2 Time windows analysis

We analyzed mind‐wandering of different time windows to track the dynamic change of MW over time. ANOVA was used to compare differences between time windows.

In terms of Mean RTs, the results showed that there were significant differences between the six time windows (Welch's *F* (5, 15431.53) = 77.66, *p* < .001, *η_p_^2^* = 0.01), and post hoc comparisons showed that there was no significant difference between time windows 1 and 2 (*p =* .720), but it was greater than time windows 3 (*p* < .001), 4 (*p* < .001), 5 (*p* < .001), 6 (*p* < .001); Mean RTs for time window 2 were greater than time windows 3 (*p* < .001), 4 (*p* < .001), 5 (*p* < .001), 6 (*p* < .001); Mean RTs for time window 3 were greater than time windows 4 (*p* < .001), 5 (*p* < .001), 6 (*p* < .001); Mean RTs for time window 4 were greater than time window 5 (*p* < .001), but there was no significant difference between time windows 4 and 6 (*p =* .997), time windows 5 and 6 (*p =* .329), as detailed in Figure 3a.

In terms of RT *CV*, the results showed that there were significant differences between the six time windows (*F* (5, 995) = 2.46, *p* < .05, *η_p_^2^* = 0.01), with post hoc comparisons only finding that the RT CV for time window 1 was significantly smaller than time window 5 (*p* < .01) and time window 6 (*p* < .05) and that time window 2 was significantly smaller than time window 4 (*p* < .05) and time window 5 (*p* < .05), while the rest of the time windows were not significantly different between each other (see Figure 3b).

For d', the results indicated a significant difference (*F* (5, 971) = 19.88, *p* < .001, *η_p_^2^* = 0.09), with post hoc comparisons finding that time window 1 was significantly larger than time windows 2 (*p* < .01), 3 (*p* < .001), 4 (*p* < .001), 5 (*p* < .001), 6 (*p* < .001); time window 2 was larger than time windows 3 (*p* = .05), 4 (*p* < .001), 5 (*p* < .001), 6 (*p* < .001); and time window 3 was significantly larger than time windows 5 (*p* < .01), 6 (*p* < .01), but there was no different between time window 3 and time window 4 (*p* = .089), time window 4 and time windows 5 (*p* = .088), time windows 4 and 6 (*p* = .086), time window 5 and time window 6 (*p* = .849) (see Figure 3c).

Those suggested that the extent of participants' MW varied over time. To explore the interaction between the time window and the degree of mind‐wandering for each dependent variable, we conducted a two‐factor ANOVA. More detailed data were provided in Supplementary Material 1.

For Mean RTs: because of the heterogeneity of variance, we conducted a two‐factor nonparametric ANOVA. Using the Scheirer‐Ray‐Hare test, the main effect of time windows was significant (Scheirer et al., 1976), *H*(5, 41497) = 62.48, *p* < .001, indicating that Mean RTs differed across time windows; there was a significant difference between Mean RTs of participants with different levels of mind‐wandering, *H*(2, 41497) = 281.48, *p* < .001, but the interaction between time windows and level of mind‐wandering was not significant, *H*(10, 41497) = 8.77, *p* = .554 (see Figure 4a).

For RT *CV*: the results showed that the main effect of time windows was not significant, *F*(5, 1001) = 1.51, *p* = .184, indicating that there was no significant difference in RT *CV* across time windows; the main effect of level of mind‐wandering was significant *F*(2, 1001) = 74.78, *p* < .001, which indicated that there was a significant difference in RT *CV* across participants with different levels of mind‐wandering, while the interaction between time and level of mind‐wandering was not significant, *F*(10, 1001) = 0.73, *p* = .693 (see Figure 4b).

For d‐prime: the main effect of time windows was significant, *F*(5, 977) = 13.38, *p* < .001; the main effect of level of mind‐wandering was significant, *F*(2, 977) = 63.76, *p* < .001; the interaction between time and level of mind‐wandering was not significant, *F*(10, 977) = 0.32, *p* = .977 (see Figure 4c).

Combining the results of the mind‐wandering analysis, we found that severe group (deepest reached modes 7–9) performed worse than individuals with moderate group (deepest reached modes 4–6) and mild group (deepest reached modes 1–3) in all aspects, suggesting that mSART was able to distinguish individuals with severe MW from other individuals. In contrast, participants with moderate and mild mind‐wandering did not differ in RT *CV*, d'.

### 3.3 “b” key

A total of 51 people used the b key, with 84% of the keystrokes after the no‐go response and 16% of the keystrokes after the go stimulus error response. This indicates that the vast majority of participants who pressed the b key chose the error correction response after the no‐go response.

## 4 DISCUSSION

Based on previous paradigms, we modified the SART paradigm in three aspects: (1) not fixing the presentation probability of no‐go stimuli; (2) adding “b” as an error‐correcting key; and (3) the fixed length of time for the mSART paradigm. We calculated Mean RTs, RT *CV*, d', and deepest mode reached to understand MW and explored the optimal length of time to induce MW through different time windows.

The results of the mind‐wandering analysis showed that 93% reached deepest into mode 4 and above; Mean RTs, RT *CV*, and d' were significantly different among the three groups (Mild: deepest reached modes 1–3; Moderate: deepest reached modes 4–6; Severe: deepest reached modes 7–9), and participants who were severely mind‐wandering performed worse than the other groups in all indicators, which was consistent with our hypothesis. Mean RTs and RT *CV* suggested that participants who deepest reached mode seven and above have greater reaction time variability, that is, deeper mind‐wandering. In addition, our analysis of d' revealed that severe group was significantly smaller than mild and moderate groups, but there was no significant difference between mild and moderate groups. The mild and moderate groups were better able to identify no‐go stimuli from many go stimuli than the severe group, which is consistent with the hypothesis. However, there was no significant difference in d' between the mild and moderate groups, suggesting that there may be no difference in the ability to identify no‐go stimuli from many go stimuli between the moderate and mild groups based on deepest reached mode, which is inconsistent with the hypothesis, and this may be related to the fact that the number of mild participants was much smaller than the other two groups (7% of the total). This result suggested that there were differences in the degree of participants' mind‐wandering and that mSART could distinguish participants who were severely mind‐wandering from the whole, but whether there were differences between the mild group (deepest into modes 1–3) and the moderate group (deepest into modes 4–6) still needs further investigation.

We also analyzed mind‐wandering in different time windows. It was found that over the six time windows, participants in the severe group had greater RT variability compared to the moderate and mild groups, indicating deeper mind‐wandering. At the same time, the participants in the severe group mind‐wander more with increasing RT variability over time, while the mild and moderate groups’ RT variability fluctuated only slightly across time windows. The d' value was getting lower with time, which indicated a dip in performance, which may have been caused by an increase in MW, among other potential factors (such as fatigue). Severe group's d' value was lower than the other groups in all time windows, while no significant differences were seen between the mild and moderate groups. Interestingly, we found some differences in the participants' task performance between time windows 1 and 4. However, no differences in time windows 5 and 6, and we speculate that the changes in participants' performance on the mSART task may have leveled off after 20 min of the experiment. In addition, we conducted a follow‐up interview with the participants at the end of the experiment and found that they reported irritability and other emotions after 25 min. Therefore, based on the results of the behavioral experiment and the results of the participants' follow‐up interview, we suggested about 20 min as the optimal length of time to induce MW. It is worth noting that MW states only vary in severity, there is no optimum state, and the optimum duration is only considered for practical reasons. MW may be plateaus after several time windows, so exceeding this length may not be necessary for researchers interested in the dynamics of MW.

A “b” key option was used by 51 participants, and these repeated error‐correcting behaviors were almost all keystroke errors immediately following the no‐go stimuli. We assumed that participants occasionally became aware of their errors and attempted to correct them. In addition, we found that the severe group had about the same number of participants as the moderate group, while the severe participants pressed the b key much less often than the moderate participants, suggesting that the b key may also be a performance monitoring measure.

This study also had some limitations. First, we only validated our classification system against other performance‐based measures of MW such as RT *CV* but lack other valid measures independent of SART to demonstrate differences between groups, such as retrospective questionnaires. Second, the concept of mind‐wandering is broad, and this study only applies to mind‐wandering induced during the SART task. It is worth mentioning that reduced mind‐wandering does not indicate that participants are completely removed from all mind‐wandering states, as the relationship between MW and task states is not a zero‐sum game, nor can one easily be inferred from the other (Schubert et al., 2020; Weinstein et al., 2018a). Third, the current research is a preliminary exploratory study of mSART, and we only establish its feasibility. In the following studies, neurocognitive science methods (such as EEG and fNIRS) and other objective indicators should be considered to verify the structural validity of the mSART more objectively and better explore the mechanism behind the mind‐wandering. Furthermore, although we present a novel way of assessing mind‐wandering propensity, it is also very important to understand the nature of these and why they are triggered. For example, although Seli et al. (2016b), and more recently Martínez‐Pérez et al. (2021), have highlighted the practical value of distinguishing between unintentional and intentional mind‐wandering, our current paradigm cannot make such a distinction.

## 5 CONCLUSION

Regardless of the limitations of this study, mSART not only did not change the simple structure of the original paradigm, applicable to participants with lower cognitive levels but also effectively captured mind‐wandering over time. The result showed that mSART could quantify the extent of MW based on changes in the frequency at which the no‐go stimuli were presented and also revealed that the recommended length of the experiment was about 20 min.

## AUTHOR CONTRIBUTIONS

All authors contributed substantially and according to the guidelines to be recognized as authors. Study concept and design: QZ. Data acquisition: QD and YH. Identification and quality assessment of studies: YQL, LJZ, and YH. Data analysis and interpretation: YQL, QD, HYC, and LJZ. Manuscript preparation and manuscript editing: YQL, QZ, and CYZ. All authors have read and approved the final version of the manuscript.

## CONFLICT OF INTEREST STATEMENT

The authors have no relevant financial or nonfinancial interests to disclose.

### CONSENT TO PARTICIPATE

All participants voluntarily agreed to participate in the research.

### CONSENT FOR PUBLICATION

All authors have understood and agreed to publish.

### PEER REVIEW

The peer review history for this article is available at https://publons.com/publon/10.1002/brb3.3175.

## Supporting information

Supplementary Material 1

Supplementary Material 2

## Data Availability

The datasets generated during and/or analyzed during the current study are available from the corresponding author on reasonable request. Full data for details, see https://www.researchgate.net/publication/359337637_result_data.
