# Supplementary material for: Examining the effects of a modified SART when measuring mind‐wandering
Source: Brain Behav. 2023 Jul 20;13(9):e3175. doi: 10.1002/brb3.3175 (PMC10498071; doi:10.1002/brb3.3175)
Supplement: Supplementary file 1 — Supplementary Material 1 [file BRB3-13-e3175-s001.docx]

目录

[一、Descriptive Statistics 2](#_Toc138017121)

[1. The overall status of mean RTs, d-prime, and RT *CV* of participants with different levels of mind-wandering at different time windows 2](#_Toc138017122)

[1.1 Mean RTs 2](#_Toc138017123)

[1.2 d-prime 3](#_Toc138017124)

[1.3 RT CV 4](#_Toc138017125)

[2. Statistical results of mean RTs, d-prime, and RT *CV* descriptions for participants with different levels of mind-wandering 5](#_Toc138017126)

[2.1 Mean RTs 5](#_Toc138017127)

[2.2 d-prime 5](#_Toc138017128)

[2.3 RT *CV* 6](#_Toc138017129)

[3. Statistical results of mean RTs, d-prime, and RT *CV* descriptions of participants under different time windows 6](#_Toc138017130)

[3.1 Mean RTs 6](#_Toc138017131)

[3.2 d-prime 7](#_Toc138017132)

[3.3 RT *CV* 7](#_Toc138017133)

[二、One-way ANOVA 8](#_Toc138017134)

[1. Analysis of variance results of mean RTs, d-prime, and RT CV of participants with different levels of mind-wandering 8](#_Toc138017135)

[1.1 Mean RTs 8](#_Toc138017136)

[1.2 d-prime 9](#_Toc138017137)

[1.3 RT *CV* 11](#_Toc138017138)

[2. Analysis of variance results of participants' mean RTs, d-prime, and RT *CV* under different time windows 12](#_Toc138017139)

[2.1 Mean RTs 12](#_Toc138017140)

[2.2 d-prime 14](#_Toc138017141)

[2.3 RT *CV* 16](#_Toc138017142)

[三、Two-way analysis of variance was conducted to examine the interaction between time windows and levels of mind-wandering on the dependent variables, namely mean RT, d-prime, and RT CV. 18](#_Toc138017143)

[1. Mean RTs 18](#_Toc138017144)

[2. d-prime 20](#_Toc138017145)

[3. RT *CV* 21](#_Toc138017146)

[四、Scatter plot between the error rate of participants' no-go stimuli and the deepest mode reached 22](#_Toc138017147)

[五、The proportion of “b” key in different time windows 22](#_Toc138017148)

[六、T test of the difference between mean RTs preceding successful no-go stimuli and mean RTs preceding failed no-go stimuli 23](#_Toc138017149)

# 一、Descriptive Statistics

## 1. The overall status of mean RTs, d-prime, and RT *CV* of participants with different levels of mind-wandering at different time windows

### 1.1 Mean RTs

| **Report** | | | | | |
| --- | --- | --- | --- | --- | --- |
| Mean RTs |  |  |  |  |  |
| MW | Time Window | Mean | N(sample size) | Std. Deviation | Std. Error of Mean |
| 1.00(mild) | 1(1-5 min) | 351.2920 | 572 | 54.77191 | 2.29013 |
|  | 2(5-10 min) | 342.5816 | 588 | 64.57927 | 2.66320 |
|  | 3(10-15 min) | 339.2160 | 588 | 65.90093 | 2.71771 |
|  | 4(15-20 min) | 325.8531 | 640 | 66.95630 | 2.64668 |
|  | 5(20-25 min) | 321.0191 | 524 | 76.07212 | 3.32323 |
|  | 6(25-30 min) | 329.5486 | 144 | 67.11173 | 5.59264 |
|  | Total | 335.7497 | 3056 | 66.72204 | 1.20696 |
| 2.00(moderate) | 1(1-5 min) | 335.3578 | 3823 | 59.79107 | 0.96702 |
|  | 2(5-10 min) | 338.2722 | 4097 | 62.71676 | 0.97983 |
|  | 3(10-15 min) | 331.0773 | 3896 | 63.14056 | 1.01158 |
|  | 4(15-20 min) | 326.3970 | 3756 | 66.57895 | 1.08636 |
|  | 5(20-25 min) | 320.0178 | 3380 | 68.40826 | 1.17666 |
|  | 6(25-30 min) | 327.1024 | 1612 | 66.21833 | 1.64928 |
|  | Total | 330.3223 | 20564 | 64.52604 | 0.44997 |
| 3.00(severe) | 1(1-5 min) | 315.2607 | 3564 | 64.69470 | 1.08368 |
|  | 2(5-10 min) | 316.6578 | 3764 | 71.57824 | 1.16669 |
|  | 3(10-15 min) | 306.2757 | 3620 | 78.51252 | 1.30492 |
|  | 4(15-20 min) | 299.9038 | 3336 | 81.33477 | 1.40820 |
|  | 5(20-25 min) | 296.0104 | 2585 | 87.86604 | 1.72819 |
|  | 6(25-30 min) | 291.1577 | 1008 | 90.53615 | 2.85162 |
|  | Total | 306.7271 | 17877 | 77.76182 | 0.58159 |
| Total | 1(1-5 min) | 327.5036 | 7959 | 62.79777 | 0.70391 |
|  | 2(5-10 min) | 328.9430 | 8449 | 67.83377 | 0.73798 |
|  | 3(10-15 min) | 320.5891 | 8104 | 71.78902 | 0.79746 |
|  | 4(15-20 min) | 314.9214 | 7732 | 74.48819 | 0.84711 |
|  | 5(20-25 min) | 310.5349 | 6489 | 78.21978 | 0.97102 |
|  | 6(25-30 min) | 314.1212 | 2764 | 77.97592 | 1.48317 |
|  | Total | 320.5571 | 41497 | 71.71181 | 0.35203 |

### 1.2 d-prime

| **Report** | | | | | |
| --- | --- | --- | --- | --- | --- |
| d-prime |  |  |  |  |  |
| MW | Time Window | Mean | N(sample size) | Std. Deviation | Std. Error of Mean |
| 1.00(mild) | 1(1-5 min) | 2.2328 | 13 | 0.61722 | 0.17119 |
|  | 2(5-10 min) | 1.9304 | 13 | 0.82424 | 0.22860 |
|  | 3(10-15 min) | 1.7691 | 13 | 0.69464 | 0.19266 |
|  | 4(15-20 min) | 1.6002 | 13 | 0.76138 | 0.21117 |
|  | 5(20-25 min) | 1.4870 | 13 | 1.12438 | 0.31185 |
|  | 6(25-30 min) | 1.5285 | 9 | 0.81248 | 0.27083 |
|  | Total | 1.7704 | 74 | 0.83569 | 0.09715 |
| 2.00(moderate) | 1(1-5 min) | 2.0451 | 85 | 0.62374 | 0.06765 |
|  | 2(5-10 min) | 1.8027 | 83 | 0.64205 | 0.07047 |
|  | 3(10-15 min) | 1.7069 | 86 | 0.63359 | 0.06832 |
|  | 4(15-20 min) | 1.5950 | 85 | 0.66423 | 0.07205 |
|  | 5(20-25 min) | 1.5009 | 81 | 0.64271 | 0.07141 |
|  | 6(25-30 min) | 1.4330 | 58 | 0.64999 | 0.08535 |
|  | Total | 1.6957 | 478 | 0.66992 | 0.03064 |
| 3.00(severe) | 1(1-5 min) | 1.5944 | 79 | 0.56597 | 0.06368 |
|  | 2(5-10 min) | 1.4639 | 76 | 0.63971 | 0.07338 |
|  | 3(10-15 min) | 1.2518 | 79 | 0.55609 | 0.06256 |
|  | 4(15-20 min) | 1.1259 | 79 | 0.60147 | 0.06767 |
|  | 5(20-25 min) | 0.9329 | 69 | 0.68171 | 0.08207 |
|  | 6(25-30 min) | 0.9292 | 43 | 0.59840 | 0.09126 |
|  | Total | 1.2456 | 425 | 0.65170 | 0.03161 |
| Total | 1(1-5 min) | 1.8577 | 177 | 0.64195 | 0.04825 |
|  | 2(5-10 min) | 1.6627 | 172 | 0.67629 | 0.05157 |
|  | 3(10-15 min) | 1.5094 | 178 | 0.64443 | 0.04830 |
|  | 4(15-20 min) | 1.3860 | 177 | 0.68212 | 0.05127 |
|  | 5(20-25 min) | 1.2593 | 163 | 0.75594 | 0.05921 |
|  | 6(25-30 min) | 1.2439 | 110 | 0.68713 | 0.06551 |
|  | Total | 1.5055 | 977 | 0.71311 | 0.02281 |

### 1.3 RT CV

| **Report** | | | | | |
| --- | --- | --- | --- | --- | --- |
| RT *CV* |  |  |  |  |  |
| MW | TimeWindow | Mean | N(sample size) | Std. Deviation | Std. Error of Mean |
| 1.00(mild) | 1(1-5 min) | 0.1511 | 13 | 0.0243288 | 0.00675 |
|  | 2(5-10 min) | 0.1480 | 13 | 0.0312730 | 0.00867 |
|  | 3(10-15 min) | 0.1544 | 13 | 0.0319796 | 0.00887 |
|  | 4(15-20 min) | 0.1534 | 13 | 0.0390419 | 0.01083 |
|  | 5(20-25 min) | 0.1576 | 13 | 0.0327405 | 0.00908 |
|  | 6(25-30 min) | 0.1580 | 9 | 0.0396536 | 0.01322 |
|  | Total | 0.1535 | 74 | 0.0322095 | 0.00374 |
| 2.00(moderate) | 1(1-5 min) | 0.1587 | 87 | 0.0248993 | 0.00267 |
|  | 2(5-10 min) | 0.1605 | 87 | 0.0255146 | 0.00274 |
|  | 3(10-15 min) | 0.1592 | 87 | 0.0260593 | 0.00279 |
|  | 4(15-20 min) | 0.1630 | 87 | 0.0261335 | 0.00280 |
|  | 5(20-25 min) | 0.1633 | 83 | 0.0279264 | 0.00307 |
|  | 6(25-30 min) | 0.1617 | 62 | 0.0282594 | 0.00359 |
|  | Total | 0.1610 | 493 | 0.0263140 | 0.00119 |
| 3.00(severe) | 1(1-5 min) | 0.1763 | 79 | 0.0264078 | 0.00297 |
|  | 2(5-10 min) | 0.1726 | 79 | 0.0278707 | 0.00314 |
|  | 3(10-15 min) | 0.1830 | 79 | 0.0281237 | 0.00316 |
|  | 4(15-20 min) | 0.1879 | 79 | 0.0293441 | 0.00330 |
|  | 5(20-25 min) | 0.1874 | 74 | 0.0353582 | 0.00411 |
|  | 6(25-30 min) | 0.1876 | 44 | 0.0360656 | 0.00544 |
|  | Total | 0.1820 | 434 | 0.0306584 | 0.00147 |
| Total | 1(1-5 min) | 0.1659 | 179 | 0.0271071 | 0.00203 |
|  | 2(5-10 min) | 0.1649 | 179 | 0.0278828 | 0.00208 |
|  | 3(10-15 min) | 0.1694 | 179 | 0.0298889 | 0.00223 |
|  | 4(15-20 min) | 0.1733 | 179 | 0.0313956 | 0.00235 |
|  | 5(20-25 min) | 0.1734 | 170 | 0.0339198 | 0.00260 |
|  | 6(25-30 min) | 0.1713 | 115 | 0.0345678 | 0.00322 |
|  | Total | 0.1696 | 1001 | 0.0307545 | 0.0009721 |

## 2. Statistical results of mean RTs, d-prime, and RT *CV* descriptions for participants with different levels of mind-wandering

### 2.1 Mean RTs

| **Descriptives** | | | | | | | | |
| --- | --- | --- | --- | --- | --- | --- | --- | --- |
| RT |  |  |  |  |  |  |  |  |
|  | N (sample size) | Mean | Std. Deviation | Std. Error | 95% Confidence Interval for Mean |  | Minimum | Maximum |
| MW |  |  |  |  | Lower Bound | Upper Bound |  |  |
| 1.00(mild) | 3056 | 335.7497 | 66.7220 | 1.2070 | 333.3831 | 338.1162 | 7.00 | 499.00 |
| 2.00(moderate) | 20564 | 330.3223 | 64.5260 | 0.4500 | 329.4403 | 331.2042 | 4.00 | 499.00 |
| 3.00(severe) | 17877 | 306.7271 | 77.7618 | 0.5816 | 305.5871 | 307.8671 | 2.00 | 499.00 |
| Total | 41497 | 320.5571 | 71.7118 | 0.3520 | 319.8671 | 321.2471 | 2.00 | 499.00 |

### 2.2 d-prime

| **Descriptives** | | | | | | | | |
| --- | --- | --- | --- | --- | --- | --- | --- | --- |
| d-prime |  |  |  |  |  |  |  |  |
|  | N (sample size) | Mean | Std. Deviation | Std. Error | 95% Confidence Interval for Mean |  | Minimum | Maximum |
| MW |  |  |  |  | Lower Bound | Upper Bound |  |  |
| 1.00(mild) | 13 | 1.7654 | 0.7043 | 0.1953 | 1.3398 | 2.1910 | 0.89 | 3.40 |
| 2.00(moderate) | 87 | 1.6939 | 0.5438 | 0.0583 | 1.5780 | 1.8098 | 0.36 | 2.95 |
| 3.00(severe) | 79 | 1.2124 | 0.4519 | 0.0508 | 1.1112 | 1.3136 | 0.45 | 2.04 |
| Total | 179 | 1.4866 | 0.5708 | 0.0427 | 1.4024 | 1.5708 | 0.36 | 3.40 |

### 2.3 RT *CV*

| **Descriptives** | | | | | | | | |
| --- | --- | --- | --- | --- | --- | --- | --- | --- |
| RT *CV* |  |  |  |  |  |  |  |  |
|  | N (sample size) | Mean | Std. Deviation | Std. Error | 95% Confidence Interval for Mean |  | Minimum | Maximum |
| MW |  |  |  |  | Lower Bound | Upper Bound |  |  |
| 1.00(mild) | 74 | 0.1535 | 0.0322095 | 0.0037 | 0.146063 | 0.160988 | 0.0999 | 0.2276 |
| 2.00(moderate) | 493 | 0.1610 | 0.0263140 | 0.0012 | 0.158667 | 0.163324 | 0.0991 | 0.2607 |
| 3.00(severe) | 434 | 0.1820 | 0.0306584 | 0.0015 | 0.179116 | 0.184901 | 0.0560 | 0.3203 |
| Total | 1001 | 0.1696 | 0.0307545 | 0.0010 | 0.167647 | 0.171462 | 0.0560 | 0.3203 |

## 3. Statistical results of mean RTs, d-prime, and RT *CV* descriptions of participants under different time windows

### 3.1 Mean RTs

| **Descriptives** | | | | | |  |  |  |  |
| --- | --- | --- | --- | --- | --- | --- | --- | --- | --- |
| RT |  |  |  |  |  | |  |  |  |
|  | N (sample size) | Mean | Std. Deviation | Std. Error | 95% Confidence Interval for Mean | |  | Minimum | Maximum |
| Time Windows |  |  |  |  | Lower Bound | | Upper Bound |  |  |
| 1(1-5 min) | 7959 | 327.5036 | 62.79777 | 0.70391 | 326.1237 | | 328.8834 | 7.00 | 499.00 |
| 2(5-10 min) | 8449 | 328.9430 | 67.83377 | 0.73798 | 327.4963 | | 330.3896 | 3.00 | 499.00 |
| 3(10-15 min) | 8104 | 320.5891 | 71.78902 | 0.79746 | 319.0259 | | 322.1523 | 2.00 | 499.00 |
| 4(15-20 min) | 7732 | 314.9214 | 74.48819 | 0.84711 | 313.2608 | | 316.5819 | 3.00 | 499.00 |
| 5(20-25 min) | 6489 | 310.5349 | 78.21978 | 0.97102 | 308.6314 | | 312.4384 | 2.00 | 499.00 |
| 6(25-30 min) | 2764 | 314.1212 | 77.97592 | 1.48317 | 311.2130 | | 317.0294 | 4.00 | 499.00 |
| Total | 41497 | 320.5571 | 71.71181 | 0.352 | 319.8671 | | 321.2471 | 2.00 | 499.00 |

### 3.2 d-prime

| **Descriptives** | | | | | | | | |
| --- | --- | --- | --- | --- | --- | --- | --- | --- |
| d-prime |  |  |  |  |  |  |  |  |
|  | N (sample size) | Mean | Std. Deviation | Std. Error | 95% Confidence Interval for Mean |  | Minimum | Maximum |
|  |  |  |  |  | Lower Bound | Upper Bound |  |  |
| 1(1-5 min) | 177 | 1.8577 | 0.6421 | 0.04827 | 1.7625 | 1.9530 | -0.03 | 3.52 |
| 2(5-10 min) | 172 | 1.6627 | 0.6762 | 0.05156 | 1.5610 | 1.7645 | -0.01 | 3.72 |
| 3(10-15 min) | 178 | 1.5092 | 0.6444 | 0.04830 | 1.4139 | 1.6045 | 0.24 | 3.75 |
| 4(15-20 min) | 177 | 1.3862 | 0.6823 | 0.05128 | 1.2850 | 1.4874 | -0.08 | 3.21 |
| 5(20-25 min) | 163 | 1.2599 | 0.7560 | 0.05921 | 1.1429 | 1.3768 | -0.89 | 3.72 |
| 6(25-30 min) | 110 | 1.2439 | 0.6874 | 0.06554 | 1.1140 | 1.3738 | -0.26 | 3.59 |
| Total | 977 | 1.5056 | 0.7132 | 0.02282 | 1.4609 | 1.5504 | -0.89 | 3.75 |

### 3.3 RT *CV*

| **Descriptives** | | | | | | | | |
| --- | --- | --- | --- | --- | --- | --- | --- | --- |
| RT *CV* |  |  |  |  |  |  |  |  |
|  | N (sample size) | Mean | Std. Deviation | Std. Error | 95% Confidence Interval for Mean |  | Minimum | Maximum |
|  |  |  |  |  | Lower Bound | Upper Bound |  |  |
| 1(1-5 min) | 179 | 0.1659 | 0.0271071 | 0.00203 | 0.161931 | 0.169927 | 0.1107 | 0.2539 |
| 2(5-10 min) | 179 | 0.1649 | 0.0278828 | 0.00208 | 0.160800 | 0.169025 | 0.0991 | 0.2260 |
| 3(10-15 min) | 179 | 0.1694 | 0.0298889 | 0.00223 | 0.164943 | 0.173760 | 0.1008 | 0.2544 |
| 4(15-20 min) | 179 | 0.1733 | 0.0313956 | 0.00235 | 0.168667 | 0.177928 | 0.1136 | 0.2518 |
| 5(20-25 min) | 170 | 0.1734 | 0.0339198 | 0.00260 | 0.168218 | 0.178490 | 0.0560 | 0.3029 |
| 6(25-30 min) | 115 | 0.1713 | 0.0345678 | 0.00322 | 0.164908 | 0.177679 | 0.1007 | 0.3203 |
| Total | 1001 | 0.1696 | 0.0307545 | 0.00097 | 0.167647 | 0.171462 | 0.0560 | 0.3203 |

# 二、One-way ANOVA

## 1. Analysis of variance results of mean RTs, d-prime, and RT CV of participants with different levels of mind-wandering

### 1.1 Mean RTs

| **Test of Homogeneity of Variances** | | | | | | |
| --- | --- | --- | --- | --- | --- | --- |
|  |  | Levene Statistic | df1 | df2 | Sig. |  |
| RT | Based on Mean | 158.726 | 2 | 41494 | 0.000 |  |
|  | Based on Median | 159.937 | 2 | 41494 | 0.000 |  |
|  | Based on Median and with adjusted df | 159.937 | 2 | 39825.196 | 0.000 |  |
|  | Based on trimmed mean | 158.705 | 2 | 41494 | 0.000 |  |

| **Robust Tests of Equality of Means** | | | | | | | | | |
| --- | --- | --- | --- | --- | --- | --- | --- | --- | --- |
| RT | |  | |  | |  | |  | |
|  | | Statistic^a^ | | df1 | | df2 | | Sig. | |
| Welch | | 578.996 | | 2 | | 8606.038 | | 0.000 | |
| a. Asymptotically F distributed. | |  | |  | |  | |  | |
| **Measures of Association** | | | | | | | | |  |
|  | R | | R Squared | | Eta | | Eta Squared | |  |
| RT * MW | -0.161 | | 0.026 | | 0.169 | | 0.029 | |  |

| **Multiple Comparisons** | | | | | | | |  |
| --- | --- | --- | --- | --- | --- | --- | --- | --- |
| Dependent Variable: | RT | | | | | | | |
| (I) MW |  |  | Mean Difference (I-J) | Std. Error | Sig. | 95% Confidence Interval |  |  |
|  |  |  |  |  |  | Lower Bound | Upper Bound |  |
| Games-Howell | 1.00(mild) | 2.00(moderate) | 5.42741^*^ | 1.28811 | 0.000 | 2.4073 | 8.4475 |  |
|  |  | 3.00(severe) | 29.02259^*^ | 1.33978 | 0.000 | 25.8815 | 32.1636 |  |
|  | 2.00(moderate) | 1.00(mild) | -5.42741^*^ | 1.28811 | 0.000 | -8.4475 | -2.4073 |  |
|  |  | 3.00(severe) | 23.59518^*^ | 0.73534 | 0.000 | 21.8717 | 25.3187 |  |
|  | 3.00(severe) | 1.00(mild) | -29.02259^*^ | 1.33978 | 0.000 | -32.1636 | -25.8815 |  |
|  |  | 2.00(moderate) | -23.59518^*^ | 0.73534 | 0.000 | -25.3187 | -21.8717 |  |
| *. The mean difference is significant at the 0.05 level. |  |  |  |  |  |  |  |  |

### 1.2 d-prime

| **Test of Homogeneity of Variances** | | | | | |
| --- | --- | --- | --- | --- | --- |
|  |  | Levene Statistic | df1 | df2 | Sig. |
| d-prime | Based on Mean | 1.967 | 2 | 176 | 0.143 |
|  | Based on Median | 1.960 | 2 | 176 | 0.144 |
|  | Based on Median and with adjusted df | 1.960 | 2 | 157.876 | 0.144 |
|  | Based on trimmed mean | 2.023 | 2 | 176 | 0.135 |

| **ANOVA** | | | | | |
| --- | --- | --- | --- | --- | --- |
| d-prime |  |  |  |  |  |
|  | Sum of Squares | df | Mean Square | F | Sig. |
| Between Groups | 10.689 | 2 | 5.344 | 19.884 | 0.00 |
| Within Groups | 47.305 | 176 | 0.269 |  |  |
| Total | 57.994 | 178 |  |  |  |

| **Measures of Association** | | |
| --- | --- | --- |
|  | Eta | Eta Squared |
| d-prime * MW | 0.429 | 0.184 |

| **Multiple Comparisons** | | | | | | | |  |
| --- | --- | --- | --- | --- | --- | --- | --- | --- |
| Dependent Variable: | d-prime | | | | | | | |
| (I) MW |  |  | Mean Difference (I-J) | Std. Error | Sig. | 95% Confidence Interval |  |  |
|  |  |  |  |  |  | Lower Bound | Upper Bound |  |
| LSD | 1.00(mild) | 2.00(moderate) | 0.07148 | 0.15416 | 0.643 | -0.2328 | 0.3757 |  |
|  |  | 3.00(severe) | .55298^*^ | 0.15517 | 0.000 | 0.2467 | 0.8592 |  |
|  | 2.00(moderate) | 1.00(mild) | -0.07148 | 0.15416 | 0.643 | -0.3757 | 0.2328 |  |
|  |  | 3.00(severe) | .48150^*^ | 0.08057 | 0.000 | 0.3225 | 0.6405 |  |
|  | 3.00(severe) | 1.00(mild) | -.55298^*^ | 0.15517 | 0.000 | -0.8592 | -0.2467 |  |
|  |  | 2.00(moderate) | -.48150^*^ | 0.08057 | 0.000 | -0.6405 | -0.3225 |  |
| *. The mean difference is significant at the 0.05 level. |  |  |  |  |  |  |  |  |

### 1.3 RT *CV*

| **Test of Homogeneity of Variances** | | | | | |
| --- | --- | --- | --- | --- | --- |
|  |  | Levene Statistic | df1 | df2 | Sig. |
| RT *CV* | Based on Mean | 6.884 | 2 | 998 | 0.001 |
|  | Based on Median | 6.280 | 2 | 998 | 0.002 |
|  | Based on Median and with adjusted df | 6.280 | 2 | 975.547 | 0.002 |
|  | Based on trimmed mean | 6.833 | 2 | 998 | 0.001 |

| **Robust Tests of Equality of Means** | | | | |
| --- | --- | --- | --- | --- |
| RT *CV* |  |  |  |  |
|  | Statistic^a^ | df1 | df2 | Sig. |
| Welch | 69.927 | 2 | 198.107 | 0.000 |
| a. Asymptotically F distributed. |  |  |  |  |

| **Measures of Association** | | | |
| --- | --- | --- | --- |
|  | Eta | | Eta Squared |
| RT *CV* * MW | | 0.429 | 0.184 |

| **Multiple Comparisons** | | | | | | | |  |
| --- | --- | --- | --- | --- | --- | --- | --- | --- |
| Dependent Variable: | RT *CV* | | | | | | | |
| (I) MW |  |  | Mean Difference (I-J) | Std. Error | Sig. | 95% Confidence Interval |  |  |
|  |  |  |  |  |  | Lower Bound | Upper Bound |  |
| Games-Howell | 1.00(mild) | 2.00(moderate) | -0.0074702 | 0.0039274 | 0.144 | -0.016833 | 0.001892 |  |
|  |  | 3.00(severe) | -.0284831^*^ | 0.0040231 | 0.000 | -0.038059 | -0.018907 |  |
|  | 2.00(moderate) | 1.00(mild) | 0.0074702 | 0.0039274 | 0.144 | -0.001892 | 0.016833 |  |
|  |  | 3.00(severe) | -.0210129^*^ | 0.0018895 | 0.000 | -0.025449 | -0.016577 |  |
|  | 3.00(severe) | 1.00(mild) | .0284831^*^ | 0.0040231 | 0.000 | 0.018907 | 0.038059 |  |
|  |  | 2.00(moderate) | .0210129^*^ | 0.0018895 | 0.000 | 0.016577 | 0.025449 |  |
| *. The mean difference is significant at the 0.05 level. |  |  |  |  |  |  |  |  |

## 2. Analysis of variance results of participants' mean RTs, d-prime, and RT *CV* under different time windows

### 2.1 Mean RTs

| **Test of Homogeneity of Variances** | | | | | |
| --- | --- | --- | --- | --- | --- |
|  |  | Levene Statistic | df1 | df2 | Sig. |
| RT | Based on Mean | 27.120 | 5 | 41491 | 0.000 |
|  | Based on Median | 27.513 | 5 | 41491 | 0.000 |
|  | Based on Median and with adjusted df | 27.513 | 5 | 39934.030 | 0.000 |
|  | Based on trimmed mean | 27.078 | 5 | 41491 | 0.000 |

| **Robust Tests of Equality of Means** | | | | |
| --- | --- | --- | --- | --- |
| RT |  |  |  |  |
|  | Statistic^a^ | df1 | df2 | Sig. |
| Welch | 77.655 | 5 | 15431.527 | 0.000 |
| a. Asymptotically F distributed. |  |  |  |  |

| **Measures of Association** | | | | |
| --- | --- | --- | --- | --- |
|  | R | R Squared | Eta | Eta Squared |
| RT * TimeWindow | -0.089 | 0.008 | 0.097 | 0.009 |

| **Multiple Comparisons** | | | | | | | |  |
| --- | --- | --- | --- | --- | --- | --- | --- | --- |
| Dependent Variable: | RT | | | | | | | |
| (I) TimeWindow |  |  | Mean Difference (I-J) | Std. Error | Sig. | 95% Confidence Interval |  |  |
|  |  |  |  |  |  | Lower Bound | Upper Bound |  |
| Games-Howell | 1(1-5 min) | 2(5-10 min) | -1.43937 | 1.01985 | 0.720 | -4.3460 | 1.4672 |  |
|  |  | 3(10-15 min) | 6.91449^*^ | 1.06368 | 0.000 | 3.8829 | 9.9460 |  |
|  |  | 4(15-20 min) | 12.58222^*^ | 1.10140 | 0.000 | 9.4431 | 15.7213 |  |
|  |  | 5(20-25 min) | 16.96868^*^ | 1.19932 | 0.000 | 13.5504 | 20.3869 |  |
|  |  | 6(20-30 min) | 13.38238^*^ | 1.64173 | 0.000 | 8.7017 | 18.0631 |  |
|  | 2(5-10 min) | 1(1-5 min) | 1.43937 | 1.01985 | 0.720 | -1.4672 | 4.3460 |  |
|  |  | 3(10-15 min) | 8.35386^*^ | 1.08653 | 0.000 | 5.2572 | 11.4505 |  |
|  |  | 4(15-20 min) | 14.02159^*^ | 1.12348 | 0.000 | 10.8196 | 17.2236 |  |
|  |  | 5(20-25 min) | 18.40805^*^ | 1.21963 | 0.000 | 14.9319 | 21.8841 |  |
|  |  | 6(20-30 min) | 14.82175^*^ | 1.65663 | 0.000 | 10.0987 | 19.5448 |  |
|  | 3(10-15 min) | 1(1-5 min) | -6.91449^*^ | 1.06368 | 0.000 | -9.9460 | -3.8829 |  |
|  |  | 2(5-10 min) | -8.35386^*^ | 1.08653 | 0.000 | -11.4505 | -5.2572 |  |
|  |  | 4(15-20 min) | 5.66773^*^ | 1.16342 | 0.000 | 2.3519 | 8.9835 |  |
|  |  | 5(20-25 min) | 10.05419^*^ | 1.25651 | 0.000 | 6.4730 | 13.6354 |  |
|  |  | 6(20-30 min) | 6.46789^*^ | 1.68397 | 0.002 | 1.6670 | 11.2688 |  |
|  | 4(15-20 min) | 1(1-5 min) | -12.58222^*^ | 1.10140 | 0.000 | -15.7213 | -9.4431 |  |
|  |  | 2(5-10 min) | -14.02159^*^ | 1.12348 | 0.000 | -17.2236 | -10.8196 |  |
|  |  | 3(10-15 min) | -5.66773^*^ | 1.16342 | 0.000 | -8.9835 | -2.3519 |  |
|  |  | 5(20-25 min) | 4.38646^*^ | 1.28860 | 0.009 | 0.7138 | 8.0591 |  |
|  |  | 6(20-30 min) | 0.80016 | 1.70804 | 0.997 | -4.0693 | 5.6696 |  |
|  | 5(20-25 min) | 1(1-5 min) | -16.96868^*^ | 1.19932 | 0.000 | -20.3869 | -13.5504 |  |
|  |  | 2(5-10 min) | -18.40805^*^ | 1.21963 | 0.000 | -21.8841 | -14.9319 |  |
|  |  | 3(10-15 min) | -10.05419^*^ | 1.25651 | 0.000 | -13.6354 | -6.4730 |  |
|  |  | 4(15-20 min) | -4.38646^*^ | 1.28860 | 0.009 | -8.0591 | -0.7138 |  |
|  |  | 6(20-30 min) | -3.58630 | 1.77276 | 0.329 | -8.6400 | 1.4674 |  |
|  | 6(20-30 min) | 1(1-5 min) | -13.38238^*^ | 1.64173 | 0.000 | -18.0631 | -8.7017 |  |
|  |  | 2(5-10 min) | -14.82175^*^ | 1.65663 | 0.000 | -19.5448 | -10.0987 |  |
|  |  | 3(10-15 min) | -6.46789^*^ | 1.68397 | 0.002 | -11.2688 | -1.6670 |  |
|  |  | 4(15-20 min) | -0.80016 | 1.70804 | 0.997 | -5.6696 | 4.0693 |  |
|  |  | 5(20-25 min) | 3.58630 | 1.77276 | 0.329 | -1.4674 | 8.6400 |  |
| *. The mean difference is significant at the 0.05 level. |  |  |  |  |  |  |  |  |

### 2.2 d-prime

| **Test of Homogeneity of Variances** | | | | | |
| --- | --- | --- | --- | --- | --- |
|  |  | Levene Statistic | df1 | df2 | Sig. |
| d-prime | Based on Mean | 1.124 | 5 | 971 | 0.346 |
|  | Based on Median | 1.116 | 5 | 971 | 0.350 |
|  | Based on Median and with adjusted df | 1.116 | 5 | 949.334 | 0.350 |
|  | Based on trimmed mean | 1.121 | 5 | 971 | 0.347 |

| **ANOVA** | | | | | |
| --- | --- | --- | --- | --- | --- |
| d-prime |  |  |  |  |  |
|  | Sum of Squares | df | Mean Square | F | Sig. |
| Between Groups | 46.095 | 5 | 9.219 | 19.879 | 0.000 |
| Within Groups | 450.304 | 971 | 0.464 |  |  |
| Total | 496.399 | 976 |  |  |  |

| **Measures of Association** | | | | |
| --- | --- | --- | --- | --- |
|  | R | R Squared | Eta | Eta Squared |
| d-prime * TimeWindows | -0.299 | 0.089 | 0.305 | 0.093 |

| **Multiple Comparisons** | | | | | | | |  |
| --- | --- | --- | --- | --- | --- | --- | --- | --- |
| Dependent Variable: | d-prime | | | | | | | |
| (I) TimeWindows |  |  | Mean Difference (I-J) | Std. Error | Sig. | 95% Confidence Interval |  |  |
|  |  |  |  |  |  | Lower Bound | Upper Bound |  |
| LSD | 1(1-5 min) | 2(5-10 min) | .19501^*^ | 0.07291 | 0.008 | 0.0519 | 0.3381 |  |
|  |  | 3(10-15 min) | .34853^*^ | 0.07229 | 0.000 | 0.2067 | 0.4904 |  |
|  |  | 4(15-20 min) | .47153^*^ | 0.07239 | 0.000 | 0.3295 | 0.6136 |  |
|  |  | 5(20-25 min) | .59786^*^ | 0.07393 | 0.000 | 0.4528 | 0.7429 |  |
|  |  | 6(20-30 min) | .61383^*^ | 0.08268 | 0.000 | 0.4516 | 0.7761 |  |
|  | 2(5-10 min) | 1(1-5 min) | -.19501^*^ | 0.07291 | 0.008 | -0.3381 | -0.0519 |  |
|  |  | 3(10-15 min) | .15352^*^ | 0.07281 | 0.035 | 0.0106 | 0.2964 |  |
|  |  | 4(15-20 min) | .27652^*^ | 0.07291 | 0.000 | 0.1334 | 0.4196 |  |
|  |  | 5(20-25 min) | .40286^*^ | 0.07444 | 0.000 | 0.2568 | 0.5489 |  |
|  |  | 6(20-30 min) | .41882^*^ | 0.08314 | 0.000 | 0.2557 | 0.5820 |  |
|  | 3(10-15 min) | 1(1-5 min) | -.34853^*^ | 0.07229 | 0.000 | -0.4904 | -0.2067 |  |
|  |  | 2(5-10 min) | -.15352^*^ | 0.07281 | 0.035 | -0.2964 | -0.0106 |  |
|  |  | 4(15-20 min) | 0.12300 | 0.07229 | 0.089 | -0.0189 | 0.2649 |  |
|  |  | 5(20-25 min) | .24934^*^ | 0.07383 | 0.001 | 0.1045 | 0.3942 |  |
|  |  | 6(20-30 min) | .26530^*^ | 0.08259 | 0.001 | 0.1032 | 0.4274 |  |
|  | 4(15-20 min) | 1(1-5 min) | -.47153^*^ | 0.07239 | 0.000 | -0.6136 | -0.3295 |  |
|  |  | 2(5-10 min) | -.27652^*^ | 0.07291 | 0.000 | -0.4196 | -0.1334 |  |
|  |  | 3(10-15 min) | -0.12300 | 0.07229 | 0.089 | -0.2649 | 0.0189 |  |
|  |  | 5(20-25 min) | 0.12634 | 0.07393 | 0.088 | -0.0187 | 0.2714 |  |
|  |  | 6(20-30 min) | 0.14231 | 0.08268 | 0.086 | -0.0199 | 0.3046 |  |
|  | 5(20-25 min) | 1(1-5 min) | -.59786^*^ | 0.07393 | 0.000 | -0.7429 | -0.4528 |  |
|  |  | 2(5-10 min) | -.40286^*^ | 0.07444 | 0.000 | -0.5489 | -0.2568 |  |
|  |  | 3(10-15 min) | -.24934^*^ | 0.07383 | 0.001 | -0.3942 | -0.1045 |  |
|  |  | 4(15-20 min) | -0.12634 | 0.07393 | 0.088 | -0.2714 | 0.0187 |  |
|  |  | 6(20-30 min) | 0.01597 | 0.08403 | 0.849 | -0.1489 | 0.1809 |  |
|  | 6(20-30 min) | 1(1-5 min) | -.61383^*^ | 0.08268 | 0.000 | -0.7761 | -0.4516 |  |
|  |  | 2(5-10 min) | -.41882^*^ | 0.08314 | 0.000 | -0.5820 | -0.2557 |  |
|  |  | 3(10-15 min) | -.26530^*^ | 0.08259 | 0.001 | -0.4274 | -0.1032 |  |
|  |  | 4(15-20 min) | -0.14231 | 0.08268 | 0.086 | -0.3046 | 0.0199 |  |
|  |  | 5(20-25 min) | -0.01597 | 0.08403 | 0.849 | -0.1809 | 0.1489 |  |
| *. The mean difference is significant at the 0.05 level. |  |  |  |  |  |  |  |  |

### 2.3 RT *CV*

| **Test of Homogeneity of Variances** | | | | | |
| --- | --- | --- | --- | --- | --- |
|  |  | Levene Statistic | df1 | df2 | Sig. |
| RT *CV* | Based on Mean | 2.258 | 5 | 995 | 0.047 |
|  | Based on Median | 2.196 | 5 | 995 | 0.053 |
|  | Based on Median and with adjusted df | 2.196 | 5 | 939.323 | 0.053 |
|  | Based on trimmed mean | 2.224 | 5 | 995 | 0.050 |
|  |  |  |  |  |  |
| **ANOVA** | | | | | |
| RT *CV* |  |  |  |  |  |
|  | Sum of Squares | df | Mean Square | F | Sig. |
| Between Groups | 0.012 | 5 | 0.002 | 2.455 | 0.032 |
| Within Groups | 0.934 | 995 | 0.001 |  |  |
| Total | 0.946 | 1000 |  |  |  |

| **Measures of Association** | | |
| --- | --- | --- |
|  | Eta | Eta Squared |
| RT *CV* * TimeWindows | 0.110 | 0.012 |

| **Multiple Comparisons** | | | | | | | |  |
| --- | --- | --- | --- | --- | --- | --- | --- | --- |
| Dependent Variable: | RT *CV* | | | | | | | |
| (I) TimeWindows |  |  | Mean Difference (I-J) | Std. Error | Sig. | 95% Confidence Interval |  |  |
|  |  |  |  |  |  | Lower Bound | Upper Bound |  |
| LSD | 1(1-5 min) | 2(5-10 min) | 0.0010163 | 0.0032391 | 0.754 | -0.005340 | 0.007373 |  |
|  |  | 3(10-15 min) | -0.0034225 | 0.0032391 | 0.291 | -0.009779 | 0.002934 |  |
|  |  | 4(15-20 min) | -.0073689^*^ | 0.0032391 | 0.023 | -0.013725 | -0.001013 |  |
|  |  | 5(20-25 min) | -.0074252^*^ | 0.0032817 | 0.024 | -0.013865 | -0.000985 |  |
|  |  | 6(20-30 min) | -0.0053644 | 0.0036621 | 0.143 | -0.012551 | 0.001822 |  |
|  | 2(5-10 min) | 1(1-5 min) | -0.0010163 | 0.0032391 | 0.754 | -0.007373 | 0.005340 |  |
|  |  | 3(10-15 min) | -0.0044388 | 0.0032391 | 0.171 | -0.010795 | 0.001917 |  |
|  |  | 4(15-20 min) | -.0083852^*^ | 0.0032391 | 0.010 | -0.014741 | -0.002029 |  |
|  |  | 5(20-25 min) | -.0084415^*^ | 0.0032817 | 0.010 | -0.014881 | -0.002002 |  |
|  |  | 6(20-30 min) | -0.0063807 | 0.0036621 | 0.082 | -0.013567 | 0.000806 |  |
|  | 3(10-15 min) | 1(1-5 min) | 0.0034225 | 0.0032391 | 0.291 | -0.002934 | 0.009779 |  |
|  |  | 2(5-10 min) | 0.0044388 | 0.0032391 | 0.171 | -0.001917 | 0.010795 |  |
|  |  | 4(15-20 min) | -0.0039464 | 0.0032391 | 0.223 | -0.010303 | 0.002410 |  |
|  |  | 5(20-25 min) | -0.0040027 | 0.0032817 | 0.223 | -0.010443 | 0.002437 |  |
|  |  | 6(20-30 min) | -0.0019419 | 0.0036621 | 0.596 | -0.009128 | 0.005244 |  |
|  | 4(15-20 min) | 1(1-5 min) | .0073689^*^ | 0.0032391 | 0.023 | 0.001013 | 0.013725 |  |
|  |  | 2(5-10 min) | .0083852^*^ | 0.0032391 | 0.010 | 0.002029 | 0.014741 |  |
|  |  | 3(10-15 min) | 0.0039464 | 0.0032391 | 0.223 | -0.002410 | 0.010303 |  |
|  |  | 5(20-25 min) | -0.0000563 | 0.0032817 | 0.986 | -0.006496 | 0.006383 |  |
|  |  | 6(20-30 min) | 0.0020045 | 0.0036621 | 0.584 | -0.005182 | 0.009191 |  |
|  | 5(20-25 min) | 1(1-5 min) | .0074252^*^ | 0.0032817 | 0.024 | 0.000985 | 0.013865 |  |
|  |  | 2(5-10 min) | .0084415^*^ | 0.0032817 | 0.010 | 0.002002 | 0.014881 |  |
|  |  | 3(10-15 min) | 0.0040027 | 0.0032817 | 0.223 | -0.002437 | 0.010443 |  |
|  |  | 4(15-20 min) | 0.0000563 | 0.0032817 | 0.986 | -0.006383 | 0.006496 |  |
|  |  | 6(20-30 min) | 0.0020608 | 0.0036998 | 0.578 | -0.005200 | 0.009321 |  |
|  | 6(20-30 min) | 1(1-5 min) | 0.0053644 | 0.0036621 | 0.143 | -0.001822 | 0.012551 |  |
|  |  | 2(5-10 min) | 0.0063807 | 0.0036621 | 0.082 | -0.000806 | 0.013567 |  |
|  |  | 3(10-15 min) | 0.0019419 | 0.0036621 | 0.596 | -0.005244 | 0.009128 |  |
|  |  | 4(15-20 min) | -0.0020045 | 0.0036621 | 0.584 | -0.009191 | 0.005182 |  |
|  |  | 5(20-25 min) | -0.0020608 | 0.0036998 | 0.578 | -0.009321 | 0.005200 |  |
| *. The mean difference is significant at the 0.05 level. |  |  |  |  |  |  |  |  |

# 三、Two-way analysis of variance was conducted to examine the interaction between time windows and levels of mind-wandering on the dependent variables, namely mean RT, d-prime, and RT CV.

## 1. Mean RTs

| **Between-Subjects Factors** | | |
| --- | --- | --- |
|  | | N |
| Time Windows | 1(1-5 min) | 7959 |
|  | 2(5-10 min) | 8449 |
|  | 3(10-15 min) | 8104 |
|  | 4(15-20 min) | 7732 |
|  | 5(20-25 min) | 6489 |
|  | 6(25-30 min) | 2764 |
| MW | 1.00(mild) | 3056 |
|  | 2.00(moderate) | 20564 |
|  | 3.00(severe) | 17877 |

| **Levene's Test of Equality of Error Variances^a,b^** | | | | | |
| --- | --- | --- | --- | --- | --- |
|  | | Levene Statistic | df1 | df2 | Sig. |
| RT | Based on Mean | 32.939 | 17 | 41479 | .000 |
|  | Based on Median | 32.901 | 17 | 41479 | .000 |
|  | Based on Median and with adjusted df | 32.901 | 17 | 38083.226 | .000 |
|  | Based on trimmed mean | 32.622 | 17 | 41479 | .000 |
| Tests the null hypothesis that the error variance of the dependent variable is equal across groups. | | | | | |
| a. Dependent variable: RT | | | | | |
| b. Design: Intercept + Time Windows + MW + Time Windows * MW | | | | | |

| **Tests of Between-Subjects Effects** | | | | | |
| --- | --- | --- | --- | --- | --- |
| Dependent Variable: Rank of RT | | | | | |
| Source | Type III Sum of Squares(SS) | df | Mean Square | F | Sig. |
| Corrected Model | 236888668099.789^a^ | 17 | 13934627535.282 | 101.087 | .000 |
| Intercept | 7341226934502.896 | 1 | 7341226934502.896 | 53256.110 | .000 |
| Time Windows | 35863298242.806 | 5 | 7172659648.561 | 52.033 | .000 |
| MW | 161572418001.490 | 2 | 80786209000.745 | 586.055 | .000 |
| Time Windows * MW | 5035242041.934 | 10 | 503524204.193 | 3.653 | .000 |
| Error | 5717780601484.189 | 41479 | 137847600.026 |  |  |
| Total | 23819999248081.000 | 41497 |  |  |  |
| Corrected Total | 5954669269583.979 | 41496 |  |  |  |
| a. R Squared = .040 (Adjusted R Squared = .039) | | | | | |

**Scheirer-Ray-Hare analysis of variance table**

| Source of variation | SS | v（df） | s2 | H | P |
| --- | --- | --- | --- | --- | --- |
| Time Windows | 35863298242.806 | 5 |  | 62.47772  (SS_Time Windows_ / s^2^_Total_) | .000 |
| MW | 161572418001.490 | 2 |  | 281.4765  (SS_MW_ / s^2^_Total_) | .000 |
| Time Windows * MW | 5035242041.934 | 10 |  | 8.771933  (SS_Time Windows * MW_ / s^2^_Total_) | .554 |
| Error | 5717780601484.190 | 41479 |  |  |  |
| Total | 23819999248081.000 | 41497 | 574017380.7  (SS_Total_ / v_Total_) |  |  |

## 2. d-prime

| **Between-Subjects Factors** | | | |
| --- | --- | --- | --- |
|  | | N | |
| Time Windows | 1(1-5 min) | | 177 |
|  | 2(5-10 min) | | 172 |
|  | 3(10-15 min) | | 178 |
|  | 4(15-20 min) | | 177 |
|  | 5(20-25 min) | | 163 |
|  | 6(25-30 min) | | 110 |
| MW | 1.00(mild) | | 74 |
|  | 2.00(moderate) | | 478 |
|  | 3.00(severe) | | 425 |

| **Levene's Test of Equality of Error Variances^a,b^** | | | | | |
| --- | --- | --- | --- | --- | --- |
|  | | Levene Statistic | df1 | df2 | Sig. |
| d-prime | Based on Mean | 1.216 | 17 | 959 | .244 |
|  | Based on Median | 1.084 | 17 | 959 | .365 |
|  | Based on Median and with adjusted df | 1.084 | 17 | 868.392 | .365 |
|  | Based on trimmed mean | 1.237 | 17 | 959 | .228 |
| Tests the null hypothesis that the error variance of the dependent variable is equal across groups. | | | | | |
| a. Dependent variable: d-prime | | | | | |
| b. Design: Intercept + Time Windows + MW + Time Windows * MW | | | | | |

| **Tests of Between-Subjects Effects** | | | | | | | | |
| --- | --- | --- | --- | --- | --- | --- | --- | --- |
| Dependent Variable: DPRIME | | | | | | | | |
| Source | Type III Sum of Squares | df | Mean Square | F | Sig. | Partial Eta Squared | Noncent. Parameter | Observed Power^b^ |
| Corrected Model | 100.944^a^ | 17 | 5.938 | 14.403 | .000 | .203 | 244.846 | 1.000 |
| Intercept | 1179.421 | 1 | 1179.421 | 2860.754 | .000 | .749 | 2860.754 | 1.000 |
| Time Windows | 27.574 | 5 | 5.515 | 13.377 | .000 | .065 | 66.883 | 1.000 |
| MW | 52.577 | 2 | 26.288 | 63.764 | .000 | .117 | 127.528 | 1.000 |
| Time Windows * MW | 1.299 | 10 | .130 | .315 | .977 | .003 | 3.151 | .171 |
| Error | 395.373 | 959 | .412 |  |  |  |  |  |
| Total | 2710.820 | 977 |  |  |  |  |  |  |
| Corrected Total | 496.317 | 976 |  |  |  |  |  |  |
| a. R Squared = .203 (Adjusted R Squared = .189) | | | | | | | | |
| b. Computed using alpha = .05 | | | | | | | | |

## 3. RT *CV*

| **Between-Subjects Factors** | | | |
| --- | --- | --- | --- |
|  | | N | |
| Time Windows | 1(1-5 min) | | 179 |
|  | 2(5-10 min) | | 179 |
|  | 3(10-15 min) | | 179 |
|  | 4(15-20 min) | | 179 |
|  | 5(20-25 min) | | 170 |
|  | 6(25-30 min) | | 115 |
| MW | 1.00(mild) | | 74 |
|  | 2.00(moderate) | | 493 |
|  | 3.00(severe) | | 434 |

| **Levene's Test of Equality of Error Variances^a,b^** | | | | | |
| --- | --- | --- | --- | --- | --- |
|  | | Levene Statistic | df1 | df2 | Sig. |
| RTCV | Based on Mean | 1.436 | 17 | 983 | .112 |
|  | Based on Median | 1.170 | 17 | 983 | .283 |
|  | Based on Median and with adjusted df | 1.170 | 17 | 862.807 | .283 |
|  | Based on trimmed mean | 1.381 | 17 | 983 | .137 |
| Tests the null hypothesis that the error variance of the dependent variable is equal across groups. | | | | | |
| a. Dependent variable: RTCV | | | | | |
| b. Design: Intercept + Time Windows + MW + Time Windows * MW | | | | | |

| Tests of Between-Subjects Effects | | | | | | | | |
| --- | --- | --- | --- | --- | --- | --- | --- | --- |
| Dependent Variable: RTCV | | | | | | | | |
| Source | Type III Sum of Squares | df | Mean Square | F | Sig. | Partial Eta Squared | Noncent. Parameter | Observed Power^b^ |
| Corrected Model | .141^a^ | 17 | .008 | 10.118 | .000 | .149 | 172.005 | 1.000 |
| Intercept | 13.555 | 1 | 13.555 | 16552.536 | .000 | .944 | 16552.536 | 1.000 |
| Time Windows | .006 | 5 | .001 | 1.508 | .184 | .008 | 7.541 | .533 |
| MW | .122 | 2 | .061 | 74.778 | .000 | .132 | 149.556 | 1.000 |
| Time Windows * MW | .006 | 10 | .001 | .734 | .693 | .007 | 7.341 | .394 |
| Error | .805 | 983 | .001 |  |  |  |  |  |
| Total | 29.723 | 1001 |  |  |  |  |  |  |
| Corrected Total | .946 | 1000 |  |  |  |  |  |  |
| a. R Squared = .149 (Adjusted R Squared = .134) | | | | | | | | |
| b. Computed using alpha = .05 | | | | | | | | |

#
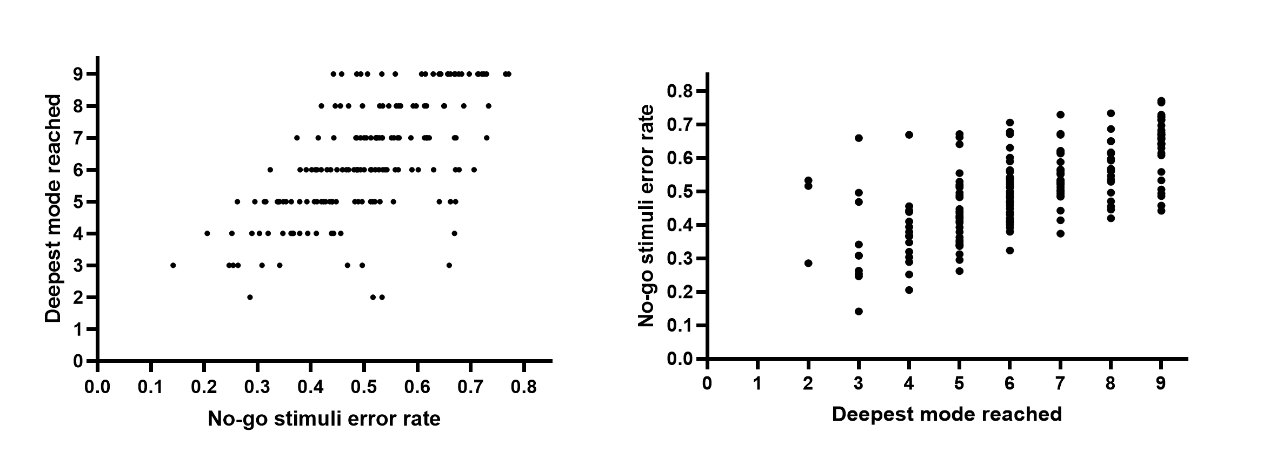
四、Scatter plot between the error rate of participants' no-go stimuli and the deepest mode reached

# 五、The proportion of “b” key in different time windows

*
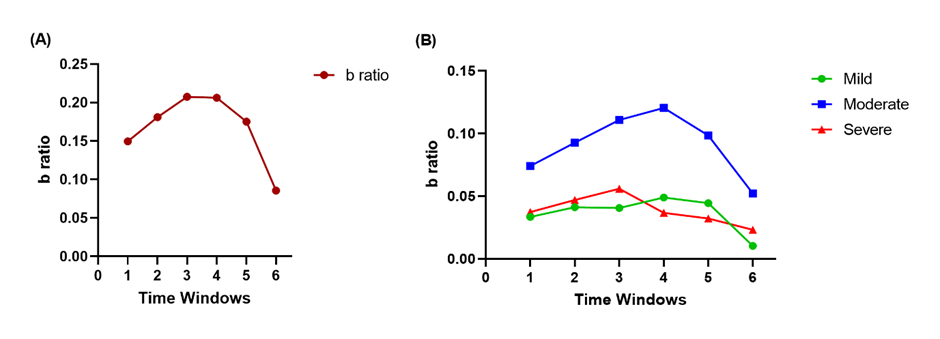
*

The proportion of “b” key in different time windows Figure (A) represents the proportion of participants' b-keys in different time windows, and Figure (B) represents the proportion of participants' b-keys in different time windows with different levels of MW.

# 六、T test of the difference between mean RTs preceding successful no-go stimuli and mean RTs preceding failed no-go stimuli

| **Group Statistics** | | | | | |
| --- | --- | --- | --- | --- | --- |
|  | 0= mean RTs preceding failed no-go stimuli; 1= mean RTs preceding successful no-go stimuli | N | Mean | Std. Deviation | Std. Error Mean |
| RT | 1.00 | 17429 | 339.9173 | 63.79458 | .48322 |
|  | .00 | 24068 | 306.5373 | 73.83552 | .47593 |

| **Independent Samples Test** | | | | | | | | | | |
| --- | --- | --- | --- | --- | --- | --- | --- | --- | --- | --- |
|  | | Levene's Test for Equality of Variances | | t-test for Equality of Means | | | | | | |
|  |  | F | Sig. | t | df | Sig. (2-tailed) | Mean Difference | Std. Error Difference | 95% Confidence Interval of the Difference | |
|  |  |  |  |  |  |  |  |  | Lower | Upper |
| RT | Equal variances assumed | 137.019 | .000 | 48.085 | 41495 | .000 | 33.38005 | .69418 | 32.01944 | 34.74066 |
|  | Equal variances not assumed |  |  | 49.215 | 40227.821 | .000 | 33.38005 | .67824 | 32.05068 | 34.70943 |

The result showed that the t-test for the difference between these two means was highly significant, t (40227.82) = 49.22, P < .001 (mean RT preceding successful no-go trials = 339.92, SE = 0.483; mean RT preceding failed no-go trials = 306.54, SE = .476). This result is consistent with the results in the article by Cheyne et al. (2006).
