# Supplementary material for: Examining the effects of a modified SART when measuring mind‐wandering
Source: Brain Behav. 2023 Jul 20;13(9):e3175. doi: 10.1002/brb3.3175 (PMC10498071; doi:10.1002/brb3.3175)
Supplement: Supplementary file 2 — Supplementary Material 2 [file BRB3-13-e3175-s002.docx]

Overall and descriptive statistics of participant b-keys, and the proportion of b-keys after go errors and no-go errors.

| Participant No. | Number of b-keys | After go error | After no-go error |
| --- | --- | --- | --- |
| 1 | 32.00 | 0 | 32 |
| 2 | 1.00 | 1 | 0 |
| 3 | 80.00 | 0 | 80 |
| 4 | 19.00 | 0 | 18 |
| 5 | 13.00 | 0 | 12 |
| 6 | 1.00 | 0 | 0 |
| 7 | 45.00 | 0 | 45 |
| 8 | 73.00 | 0 | 73 |
| 9 | 53.00 | 0 | 53 |
| 10 | 10.00 | 0 | 10 |
| 11 | 2.00 | 0 | 1 |
| 12 | 3.00 | 0 | 3 |
| 13 | 59.00 | 1 | 58 |
| 14 | 4.00 | 1 | 3 |
| 15 | 1.00 | 1 | 0 |
| 16 | 5.00 | 1 | 4 |
| 17 | 83.00 | 1 | 81 |
| 18 | 11.00 | 2 | 9 |
| 19 | 97.00 | 0 | 97 |
| 20 | 63.00 | 0 | 62 |
| 21 | 62.00 | 0 | 62 |
| 22 | 1.00 | 1 | 0 |
| 23 | 40.00 | 1 | 39 |
| 24 | 70.00 | 0 | 70 |
| 25 | 2.00 | 2 | 0 |
| 26 | 69.00 | 0 | 69 |
| 27 | 5.00 | 0 | 5 |
| 28 | 4.00 | 0 | 4 |
| 29 | 46.00 | 0 | 45 |
| 30 | 9.00 | 0 | 9 |
| 31 | 1.00 | 0 | 1 |
| 32 | 1.00 | 0 | 1 |
| 33 | 2.00 | 1 | 1 |
| 34 | 9.00 | 0 | 9 |
| 35 | 1.00 | 1 | 0 |
| 36 | 1.00 | 0 | 1 |
| 37 | 1.00 | 0 | 1 |
| 38 | 31.00 | 0 | 31 |
| 39 | 1.00 | 1 | 0 |
| 40 | 17.00 | 5 | 10 |
| 41 | 54.00 | 0 | 54 |
| 42 | 57.00 | 0 | 56 |
| 43 | 32.00 | 0 | 32 |
| 44 | 2.00 | 0 | 1 |
| 45 | 46.00 | 1 | 45 |
| 46 | 60.00 | 1 | 58 |
| 47 | 64.00 | 0 | 64 |
| 48 | 85.00 | 0 | 85 |
| 49 | 24.00 | 0 | 24 |
| 50 | 55.00 | 2 | 53 |
| 51 | 46.00 | 0 | 45 |
| Total | 1553.00 | 24 | 1516 |
| M | 30.45 | 0.47 | 29.73 |
| SD | 29.20 | 0.87 | 29.34 |
| Proportion |  | 0.02 | 0.98 |

We conducted post-hoc interviews with approximately one-tenth of the participants (i.e., 19 people), and the interview questions are detailed below. Through the interviews we focused on the effect of the b key on the participants, the changes in the participants' self-perceived wandering throughout the experiment, and their emotional state.

Regarding the b-button, most participants indicated that they did not press the b-button during the experiment because they felt that they did not press the wrong key, so they did not need the b-button. The participants who did press the b key said that they would press the b key immediately after their no-go error to correct it, and they did not feel that the requirement of the b key had an effect on completing the experimental task.

On the issue of wandering, all interviewed participants indicated that they would wander during the task, but their wandering varied. Some participants indicated that they were attentive to the task the whole time and only had occasional moments of wandering, but after that they were engaged in the task again. This process was repeated throughout the experiment. Another part of the participants said that although they were also completing the task, they were more often distracted because they found the task too easy and boring.

In terms of emotional state, the vast majority of participants indicated that they became irritable in the late stage of the experiment and wanted to end the experiment as soon as possible, while only a few participants indicated that they felt nothing.

**Main content of the interview**

1. Prelude: (personal information)

Hello, thank you for participating in our return visit survey!

Do you remember some details of participating in our "mind-wandering induced experiment"? Next, we will ask you a few questions about your psychological feelings during the experiment. Of course, this interview will follow the natural principle. If you feel uncomfortable or don't want to answer, you can refuse to answer or terminate the interview. If you have other questions, you can also communicate with me.

二．Mind-wandering degree

1. Did you lose your mind during the experiment? Can you talk about it in detail? When exactly?

2. Do you realize that the probability of no-go stimulation increases?

3. Have you ever been distracted during mind-wandering process? If so, can you tell me exactly when it happened? What is the reason? How many times did it happen? Is it because the experimental design "b" key " or target stimulation frequency"? Or for your own reasons?

4. How long do you think you were mind-wandering during this experiment? What is your estimate of the extent of the wandering?

5. Does your level of wandering deepen sequentially over time? Or does it change? For example, does it get worse and then lighter?

三．Experimental paradigm

1. Do you feel that there is self-monitoring of your consciousness in the process?

2. Do you think 30 minutes is an appropriate amount of time to induce mind-wandering? If not? How long do you think is the most appropriate?

3. Did you realize that you had pressed the wrong key when you chose to press the "b" key? Have you ever pressed the "b" key when you were distracted? Did pressing the "b" key have any effect on you?
